# Enhanced O_2_ availability in platelet concentrates stored for neonatal transfusion is independent of agitation: Evidence from direct oximetry and Fickian diffusion modelling

**DOI:** 10.1111/vox.70101

**Published:** 2025-08-28

**Authors:** Dean Pym, Oleg Grinberg, Amanda J. Davies, Jessica O. Williams, Christine Saunders, Chloë E. George, Philip E. James

**Affiliations:** ^1^ Centre of Cardiovascular Health and Ageing Cardiff Metropolitan University Cardiff UK; ^2^ Welsh Blood Service, Component Development and Research Laboratory Pontyclun UK; ^3^ Dartmouth Medical School Hanover New Hampshire USA

**Keywords:** agitation, electron paramagnetic resonance, neonatal, oxygen, platelet concentrates

## Abstract

**Background and Objectives:**

Platelet transfusions are essential for mitigating the bleeding risk of neonatal patients with thrombocytopenia. As neonatal patients have a small blood volume, adult therapeutic dose platelet units are split into reduced‐volume storage containers to maximize the use of the donated product and reduce donor exposure. The shelf‐life of platelets stored in reduced‐volume containers, however, is limited to 5 days. Agitation in platelet concentrate (PC) storage is thought to promote gaseous exchange by maintaining a gradient of O_2_ across the bag film; however, recent studies have shown that agitation‐induced shear promotes the progressive decline of platelet quality over storage.

**Materials and Methods:**

Electron paramagnetic resonance oximetry and Fickian diffusion modelling of O_2_ were used to investigate the differences in O_2_ availability, by assessing the O_2_ concentration, oxygen consumption rate (OCR), influx of O_2_, total PC OCR and O_2_ distribution in PCs stored under routine conditions in neonatal (Macopharma, VQE605B) versus adult (Haemonetics, ATSBC1ESE) PC storage containers. The influence of agitation on neonatal PC storage was evaluated.

**Results:**

Results indicate neonatal PCs experience significantly higher O_2_ availability compared to adult PCs and can withstand greater insult to their ambient O_2_ concentration. Adjusting the agitation frequency of neonatal PCs stored from 20 to 400 rpm had no detrimental effect on O_2_ availability, compared to storage at 60 rpm.

**Conclusion:**

Neonatal PCs can maintain higher O_2_ availability and tolerate reduced agitation without compromising oxygenation; therefore, reduced agitation strategies may be a feasible option to minimize shear during storage without compromising O_2_ availability.

**Highlights**

- In comparison to platelet concentrates (PCs) for adult transfusion (290–310 mL), PCs prepared for neonatal transfusion (50–70 mL) have improved O_2_ availability, when both are stored under continuous agitation for up to 8 days.
- Neonatal PCs are more resilient to reductions in ambient O_2_ during storage compared to adult PCs.
- Agitation does not influence O_2_ availability in neonatal PCs.

## INTRODUCTION

Platelet (PLT) transfusions are essential for mitigating the bleeding risk of neonatal patients with active bleeding or thrombocytopenia [1]. The highest bleeding incidence of any patient population requiring urgent PLT transfusion occurs in very low birth weight babies, with up to 25% experiencing intracranial haemorrhages [2]. Moreover, roughly1%–5% of all neonates, and up to 50% of critically ill new‐borns, are thrombocytopenic at birth [3, 4]. Because of the reduced blood volume of neonatal patients, splitting of the adult therapeutic dose (ATD or adult platelet concentrate [PC]) into reduced‐volume storage containers (neonatal PC) is done to maximize the use of the donated product and minimize donor exposure. However, current storage practices using these containers are limited to 5 days and may promote suboptimal quality compared to their adult dose counterparts [5, 6, 7].

Agitation during PC storage is thought to promote gaseous exchange by maintaining a gradient of O_2_ across the bag film [8], thus preventing the development of regions of local anoxia throughout the PC and ensuring that a sufficient PLT suspension is maintained [9]. The employment of agitation has been one of the most effective advancements in PC storage, extending PC storage from 3 days in the 1980s to the current 7‐day shelf‐life [9, 10, 11, 12]. O_2_ availability and sufficient gaseous exchange in PCs are essential in delaying the gradual deterioration of PLT viability and function during storage, by meeting the metabolic demands of the cellular suspension [11, 12, 13, 14]. Oxidative metabolism supplies around 85% of the energy generated by PLTs in storage [14]; so imbalances in the supply and demand of O_2_ can lead to morphological and functional impairments that reduce the therapeutic effectiveness of the PC in clinical applications. Insufficient O_2_ is associated with increased reliance on anaerobic glycolysis, promoting the accumulation of lactate and limiting ATP generation [15, 16]. Building on this, Nash et al. [17] reported agonist‐induced aggregation following PC storage at 10% [O_2_], underscoring the importance of maintaining adequate oxygenation to preserve PC quality.

Recent studies have highlighted agitation‐induced shear to be responsible for enhancing the progressive decline of PLT viability and function during PC storage. A study conducted by Hosseini et al. [18] showed that PLT‐rich plasma–derived PCs stored with ~70 mL autologous plasma and kept under continuous agitation displayed enhanced glycoprotein VI (GPVI) shedding and reduced PLT spreading and adhesion compared to PCs manually mixed once daily. Moreover, a study by Pym et al. [6] showed that agonist‐induced aggregation was impaired in neonatal PCs from Day 2, along with increased CD62p expression, compared to adult PCs. Adjusting the agitation frequency of neonatal PCs to 40 rpm improved agonist‐induced aggregation throughout the storage period with lower activation, compared to storage at 60 rpm [6]. The current study builds upon these findings and investigates the differences in O_2_ availability of neonatal PCs compared to adult PCs at both 21% and 5% ambient O_2_ and examines whether agitation affects O_2_ availability. Electron paramagnetic resonance (EPR) oximetry was used to measure the direct O_2_ concentration ([O_2_]) in the PC and the oxygen consumption rate (OCR). Fickian diffusion modelling using the experimental data was then used to determine the influx of O_2_ into the PC (*J*_in_), the total OCR and the distribution of O_2_ from the bag surface to the bag midpoint.

## MATERIALS AND METHODS

### PC preparation and storage

**FIGURE 1:**
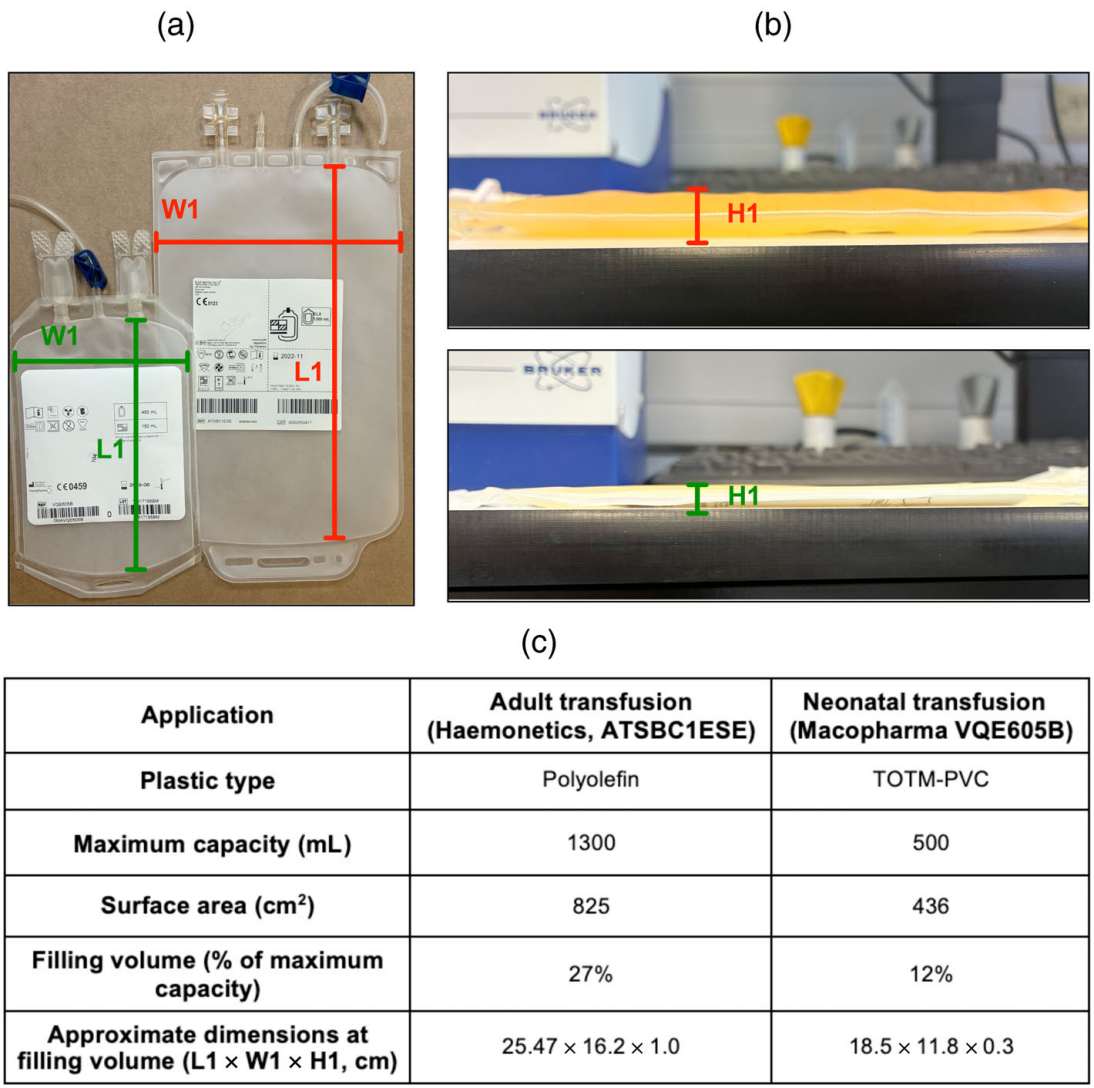
Characteristics of platelet concentrate (PC) storage bags for adult and neonatal PC storage at the Welsh Blood Service. (a) Front view of adult (right) and neonatal (left) PC storage bags showing measured length (L1) and width (W1) dimensions. (b) Side profile images of each bag when filled to the relevant working volume, showing height (H1) corresponding to fluid thickness under standard storage conditions. H1 was measured at the midpoint of the bag. (c) Summary table comparing physical and material characteristics of the two bag types, including manufacturer, plastic type, capacity, surface area, filling volume and dimensions at working volume. TOTM‐PVC, PVC plasticized with trioctyl trimellitate.

Buffy coats (BCs) were collected from whole‐blood donations using bottom and top packs from Macopharma (code: LQT614B) composed of polyvinyl chloride (PVC) plasticized with di(ethylhexyl) terephthalate (DEHP), and anticoagulated with citrate‐phosphate‐dextrose. Whole‐blood donations were kept at 22 ± 2°C without agitation overnight in accordance with the Joint United Kingdom Blood Transfusion and Tissue Transplantation Services Professional Advisory Committee (JPAC) guidelines [19]. Four ABO‐specific BCs were pooled with 250 mL of PLT additive solution (PAS) (SSP+™, Macopharma, Moureaux, France), providing a PAS‐to‐plasma ratio of approximately 65%:35%. The BC pools were centrifuged at 500*g* for 8 min, and the PLT‐rich plasma/PAS was separated from the remaining red cells using a blood component separator (CompoMat G5+, Fresenius Kabi, Germany), which simultaneously leucodepleted the PC through an integral white cell reduction filter (Autostop™; Haemonetics, Boston, MA, USA) to provide an ATD of PLTs. For each replicate experiment (*N* = 6), two ATDs were pooled and split to create two homogeneous adult dose PCs (290–310 mL). One of the two twinned ATD PCs was subsequently split into four identical neonatal PCs consisting of a volume between 50 and 70 mL. The PLT concentration of the ATD used in these studies was 1038 ± 30 × 10^9^/L. This study focuses on two PC storage containers routinely used at the Welsh Blood Service for adult and neonatal PCs, respectively, and the storage bag characteristics are shown in Figure 1. Storage followed standard blood banking conditions (22 ± 2°C with constant 60 rpm agitation with a horizontal displacement of ±2 cm). Experiments at normal ambient [O_2_], where agitation was altered between 20 and 60 rpm, were conducted to six experimental replicates (*N* = 6), while experiments where ambient storage [O_2_] was reduced using an InVivO_2_ chamber (Ruskin Ltd) were completed to three experimental replicates (*N* = 3). Experimental measures used for oxygen modelling were taken after 24 h of PC storage to allow for the establishment of O_2_ gradients under altered storage conditions while maintaining cell viability. Storage studies were undertaken over 8 days of storage.

### Electron paramagnetic resonance oximetry

EPR oximetry was employed to measure the oxygen concentration ([O_2_]) and OCR using per deuterated N^15^ Tempo (PDT; 0.2 mM; Sigma‐Aldrich). Calibration was done in PAS inside a gas‐permeable Teflon® tube placed in a quartz capillary tube exposed to either pure nitrogen (0% O_2_) or air (21% O_2_). EPR spectral line widths were used to determine [O_2_] and were calibrated under pure nitrogen (zero O_2_) and air (21% O_2_) conditions, and [O_2_] in experimental samples ([O_2_]_sample_) was calculated using Equation (1). This was referred to as ‘Direct [O_2_]’. To measure OCR, PC samples (typically containing 1 × 10^8^ platelets) with PDT were sealed in a quartz capillary tube rapidly to minimize ambient O_2_ exposure, and EPR spectra were recorded every minute for 30 min. The linear change in linewidth was converted to [O_2_] using Equation (1), with the initial linewidth reflecting [O_2_] inside the storage bag.

(1) $$\frac{{\left[O_{2}\right]}_{\text{sample}}-{\left[O_{2}\right]}_{\text{nitr}}}{{\left[O_{2}\right]}_{\mathrm{air}}-{\left[O_{2}\right]}_{\text{nitr}}\;}\times 210\;\mathrm{\mu M}.$$

### Oxygen modelling

The influx of O_2_ through the bag surface was first modelled using the conventional Fick's law equation (assuming unidirectional steady state diffusion), where *J* is the oxygen flux, ∇[O_2_] is the O_2_ gradient, *s* is the surface area (1 cm^2^), *dx* is the film thickness (cm) (Equation 2) and *D* is the diffusion coefficient of the bag. *D* was calculated from the O_2_ transmission rate of PVC plasticized with trioctyl trimellitate (TOTM‐PVC) and polyolefin, provided as ~690 and ~2000 cm^3^/m^2^/24 h, respectively. For simplicity, the bag is divided into individual parallelepipeds acting through an area of 1 cm^2^, through a length equal to the bag thickness and half the thickness of a full PC (Figure S1). It was assumed that all parallelepipeds were equal and acted identically to one another. The total OCR per parallelepiped was calculated using Equation (3), where the measured OCR is the rate of change in [O_2_] per unit (PLT), and *d* is half the bag thickness:

(2) $$J=D\;\frac{\nabla \left[O_{2}\right]}{\mathrm{dx}\times s},$$

(3) TotalOCR=MeasuredOCR×d.

It was of interest to model how closely the reported direct [O_2_] fits a homogeneous distribution. Derivation of the model can be found in Supporting Information. Briefly, a one‐dimensional steady‐state diffusion equation of oxygen concentration [O_2_] inside the bag is described by Equation (4), where *d* is half the bag thickness, O_2_ represents the oxygen concentration at a given position within the PC, OCR represents the oxygen consumption rate, *D* is the diffusion coefficient (given as *D*_water_ at 25°C = 2.42 × 10^−5^ cm^2^/s), *x* represents the distance from a certain point in the bag to the centre of the bag (excluding film thickness) and <[O_2_]> represents the direct O_2_ measured. Distribution plots were produced using RStudio [20].

(4) $$O_{2}=\frac{\mathrm{OCR}}{2*D}\times x^{2}+<\left[O_{2}\right]>-\frac{\mathrm{OCR}}{D\times 6}d^{2},$$

Using Equation (4), the total change of O_2_ throughout the PC can be calculated using the maximum and minimum deviation from <[O_2_]>, where *x* = 0 and *x* = *d* give the minimum and a maximum, respectively. By subtracting the minimum deviation from <[O_2_]> (−OCR × *d* ^2^/*D* × 6) from the maximum (OCR × *x* ^2^/2 × *D* − OCR × *d* ^2^/*D* × 6), the total variation can be simplified to Equation (5), where ∆*O*_2 surface‐midpoint_ represents the total deviation from <[O_2_]>.

(5) $$∆{O_{2}}_{\text{surface}‐\text{midpoint}}=\frac{\mathrm{OCR}}{2\times D}x^{2}-\frac{\mathrm{OCR}}{D\times 6}d^{2}--\frac{\mathrm{OCR}}{D\times 6}d^{2}=\frac{\mathrm{OCR}}{2\times D}\times d^{2}.$$

### Statistical analysis

Data analysis was carried out using GraphPad Prism 9 software (San Diego, USA). Statistical significance was inferred by a (paired or unpaired) *t*‐test or a one‐way ANOVA, followed by a post hoc Tukey's test.

## RESULTS

### O_2_ availability is improved in PCs stored for neonatal transfusion

**FIGURE 2:**
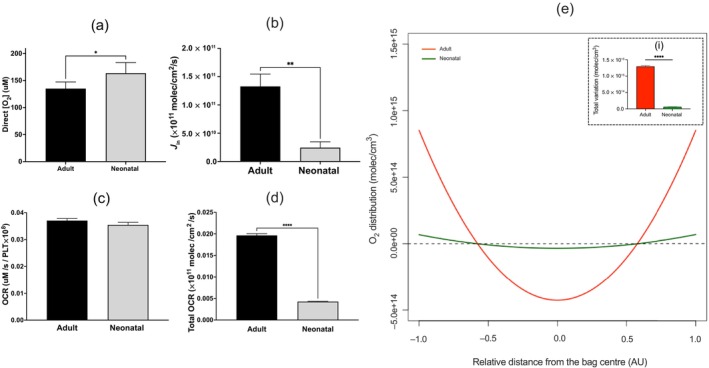
O_2_ availability in platelet concentrate (PCs) stored for adult and neonatal transfusion under standard blood banking conditions. (a) The measured [O_2_] and (b) calculated *J*_in_. (c) The oxygen consumption rate (OCR) measured by electron paramagnetic resonance (EPR) and (d) the total OCR calculated by Equation (3). (e) The O_2_ distribution, with the *X*‐axis representing the relative location in the bag in arbitrary units (AU), either the bag midpoint (0) or the bag surface (±1). The solid red line represents the O_2_ distribution for adult PCs, and the solid green line represents the O_2_ distribution for neonatal PCs. The dotted black line depicts a homogeneous O_2_ distribution. (i) shows the total deviation of O_2_ in adult and neonatal PCs. Statistical significance was inferred by a paired *t*‐test, and denoted as follows: *p < 0.05; ****p < 0.0001 (*N* = 6). PLT, platelet.

The measured [O_2_] was significantly higher in neonatal PCs (163.5 ± 44.48 μM) compared to adult PCs (135.1 ± 27.65 μM; Figure 2a). Likewise, there was a significant change in the modelled O_2_ influx in neonatal PCs compared to adult PCs (Figure 2b). There was no significant difference in the measured OCR between neonatal and adult PCs (Figure 2c); however, there was a significant difference in the modelled total O_2_ consumed per parallelepiped (total OCR) in neonatal PCs compared to adult PCs (Figure 2d).

The predicted [O_2_] distribution for neonatal PCs deviates less from a homogeneous distribution compared to adult PCs (Figure 2e). Comparing the inner edge of the bag to the midpoint, neonatal PCs deviate from homogeny by +4.23 ± 0.256 and −2.12 ± 0.128 molec × 10^13^/cm^3^, respectively, while adult PCs deviate from homogeny by +8.63 ± 0.404 and −4.32 ± 0.202 molec × 10^14^/cm^3^, respectively (Table 1). The total deviation of O_2_ across the PC was significantly higher in adult PCs than neonatal PCs (Figure 2e, panel i).

**TABLE 1:** Summary of the O_2_ distribution of adult and neonatal PCs.

PC	Ambient [O_2_] (%)	Agitation (rpm)	[O_2_]_midpoint_ × 10^14^ molec/cm^3^ (%)	[O_2_]_Surface_ × 10^14^ molec/cm^3^ (%)
Adult	21%	60	−4.32 ± 0.202 (0.49 ± 0.099%)	+8.63 ± 0.404 (0.99 ± 0.19%)
Neonatal	21%	60	−0.212 ± 0.0128 (0.031 ± 0.006%)	+0.423 ± 0.0256 (0.063 ± 0.012%)
Neonatal	21%	40	−0.221 ± 0.0219 (0.035 ± 0.0047%)	+0.442 ± 0.0438 (0.069 ± 0.009%)
Neonatal	21%	20	−0.219 ± 0.0122 (0.034 ± 0.009%)	+0.437 ± 0.0244 (0.067 ± 0.009%)
Adult	5%	60	±0.0	+6.92 ± 0.0
Neonatal	5%	60	−0.205 ± 0.0137 (0.26 ± 0.07%)	+0.410 ± 0.0273 (0.51 ± 0.13%)

*Note*: The modelled O_2_ deviation from an average measured [O_2_] either at the midpoint or surface of the bag is given. Values are reported as molecules/cm^3^ (top) or as percentage deviation from the average measured [O_2_] (bottom). Note that the percentage deviation from the average measured [O_2_] could not be calculated for adult PCs stored at 5% [O_2_]. Deviation above or below the average measured [O_2_] is represented as ‘+’ or ‘−’, respectively.

Abbreviation: PC, platelet concentrate.

### Neonatal PCs maintain an equal OCR at ambient storage [O_2_ ] of 5% compared to 21%

**FIGURE 3:**
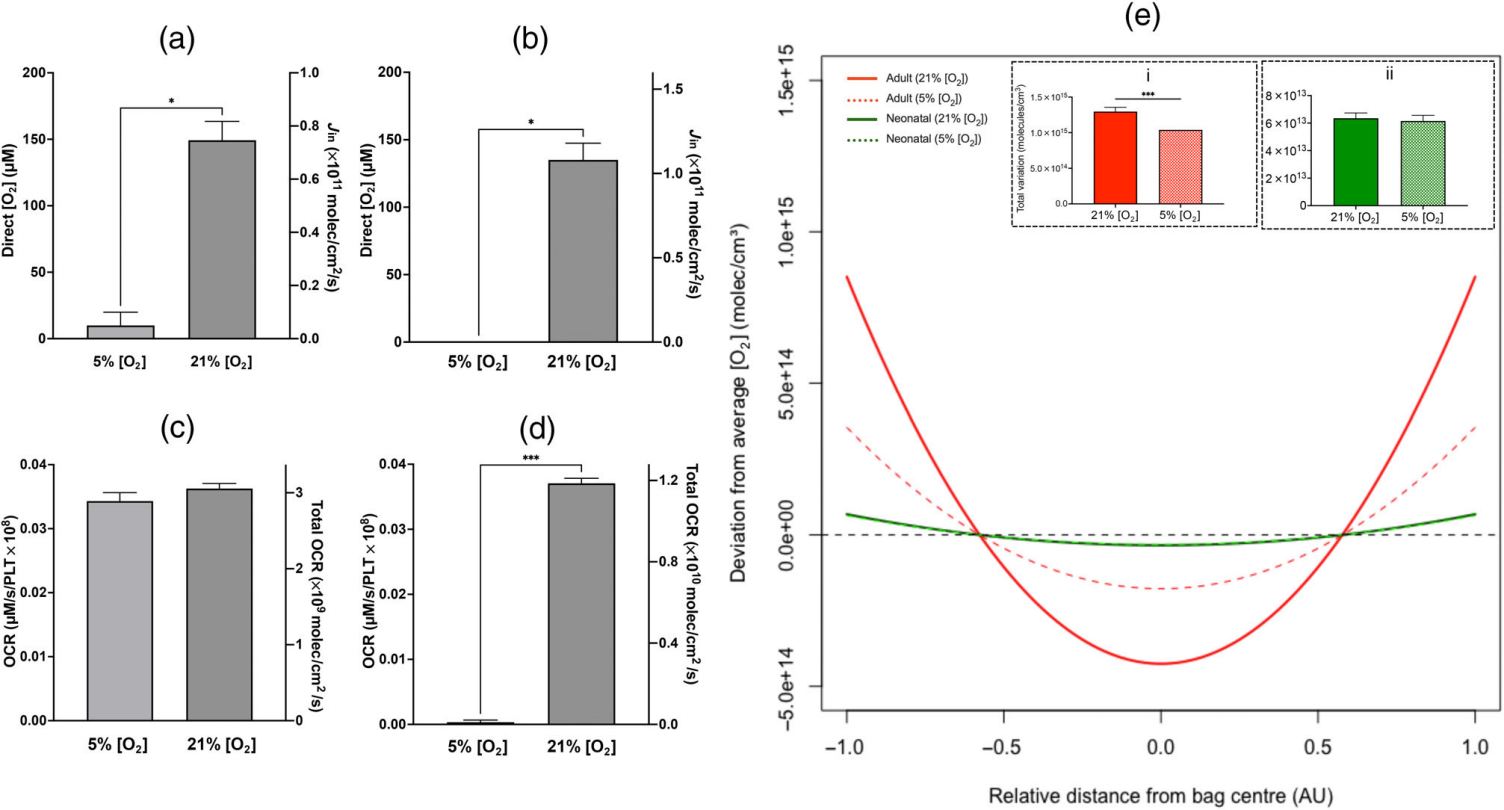
O_2_ availability in platelet concentrates (PCs) stored reduced ambient [O_2_]. (a) Measured [O_2_] and calculated *J*_in_ for neonatal PCs. (b) Measured [O_2_] and calculated *J*_in_ for adult PCs. (c) oxygen consumption rate (OCR) measured by electron paramagnetic resonance (EPR) and the total OCR calculated from Equation (3) for neonatal PCs. (d) OCR measured by EPR and the total OCR calculated from Equation (3) for adult PCs. (e) O_2_ distribution, with the *X*‐axis representing the relative location in the bag in arbitrary units (AU), either the bag midpoint (0) or the bag surface (±1). The solid red line represents the O_2_ distribution for adult PCs stored at 21% [O_2_], the solid green line represents the O_2_ distribution for neonatal PCs stored at 21% [O_2_], the dotted red line represents the O_2_ distribution for adult PCs stored at 5% [O_2_] and the dotted green line represents the O_2_ distribution for neonatal PCs stored at 5% [O_2_]. The dotted black line depicts a homogeneous O_2_ distribution. (i) The total deviation of O_2_ in adult PCs at 21% and 5% [O_2_] and (ii) the total deviation of O_2_ in neonatal PCs at 21% and 5% [O_2_]. Statistical significance was inferred by an unpaired *t*‐test, and denoted as follows: *p < 0.05; ***p < 0.001 (*N* = 3). PLT, platelet.

In neonatal PCs, the measured [O_2_] significantly decreased from 163.5 ± 44.48 μM, when stored at 21% [O_2_], to 19.9 ± 5.94 μM when stored in 5% [O_2_]. There was also a significant change in the modelled O_2_ influx following neonatal PC storage at 5% compared with 21% [O_2_] (Figure 3a). Similarly, the measured [O_2_] significantly decreased in adult PCs from 135.1 ± 27.65 μM, when stored at 21% [O_2_], to 0.0 ± 0.0 μM when stored in 5% [O_2_] (Figure 3b). There was no significant difference in OCR or the modelled total OCR between neonatal PCs stored at 5% [O_2_] and 21% [O_2_] (Figure 3c); however, OCR was significantly reduced in adult PCs stored at 5% [O_2_] compared to 21% [O_2_] (Figure 3d). In order to maintain a measured [O_2_] level within the bag equal to zero, the OCR must be equal to the influx of O_2_ into the PC (*J*_in_). Thus, assuming the total OCR = *J*_in_, OCR would be limited to 0.0297 μM/s/PLT × 10^8^. This confirms that there was a significant difference in the total OCR in adult PCs stored at 5% [O_2_] and 21% [O_2_] and in the modelled O_2_ influx.

In order to test this experimental model, we subjected both neonatal and adult PCs to low ambient O_2_ conditions. The predicted [O_2_] distribution in adult PCs stored at 5% [O_2_] appears to deviate less from a homogeneous distribution compared to PCs stored at 21% [O_2_] as the OCR was reduced from 0.0367 to 0.0297 μM/s/PLT × 10^8^ (Figure 3e). Also, the total deviation of O_2_ throughout the PC in adult PCs stored at 5% is significantly less than adult PCs stored at 21% (Figure 3e, panel i). However, it is important to note that at 5% [O_2_], the adult PC shows substantial regions of hypoxia. Using the calculated OCR for 5% [O_2_], it is predicted that adult PCs stored at 5% [O_2_] would deviate from the average measured by 6.92 ± 0.0 molec × 10^14^/cm^3^ at the bag surface and over 60% of the adult PC is in complete anoxia throughout storage. In contrast, the predicted [O_2_] distribution in neonatal PCs stored at 21% [O_2_] is equal to that of PCs stored at 5% [O_2_] (Table 1). Comparing the inner edge of the bag to the midpoint, PCs stored at 21% [O_2_] deviate from homogeny by +4.23 ± 0.256 and − 2.12 ± 0.128 molec × 10^13^/cm^3^, respectively, while neonatal PCs stored at 5% [O_2_] deviate from homogeny by +4.10 ± 0.273 and − 2.05 ± 0.137 molec × 10^13^/cm^3^, respectively. There is no significant difference in the total deviation of O_2_ in neonatal PCs stored at 21% and 5% [O_2_] (Figure 3e, panel ii) However, it is important to highlight that relative to the average measured [O_2_] in the PCs, neonatal PCs stored at 21% [O_2_] show less deviation from their average measured [O_2_] compared to neonatal PCs stored at 5% [O_2_] (Table 1).

### Agitation does not influence the O_2_ availability in neonatal PCs at 21% ambient [O_2_ ]

**FIGURE 4:**
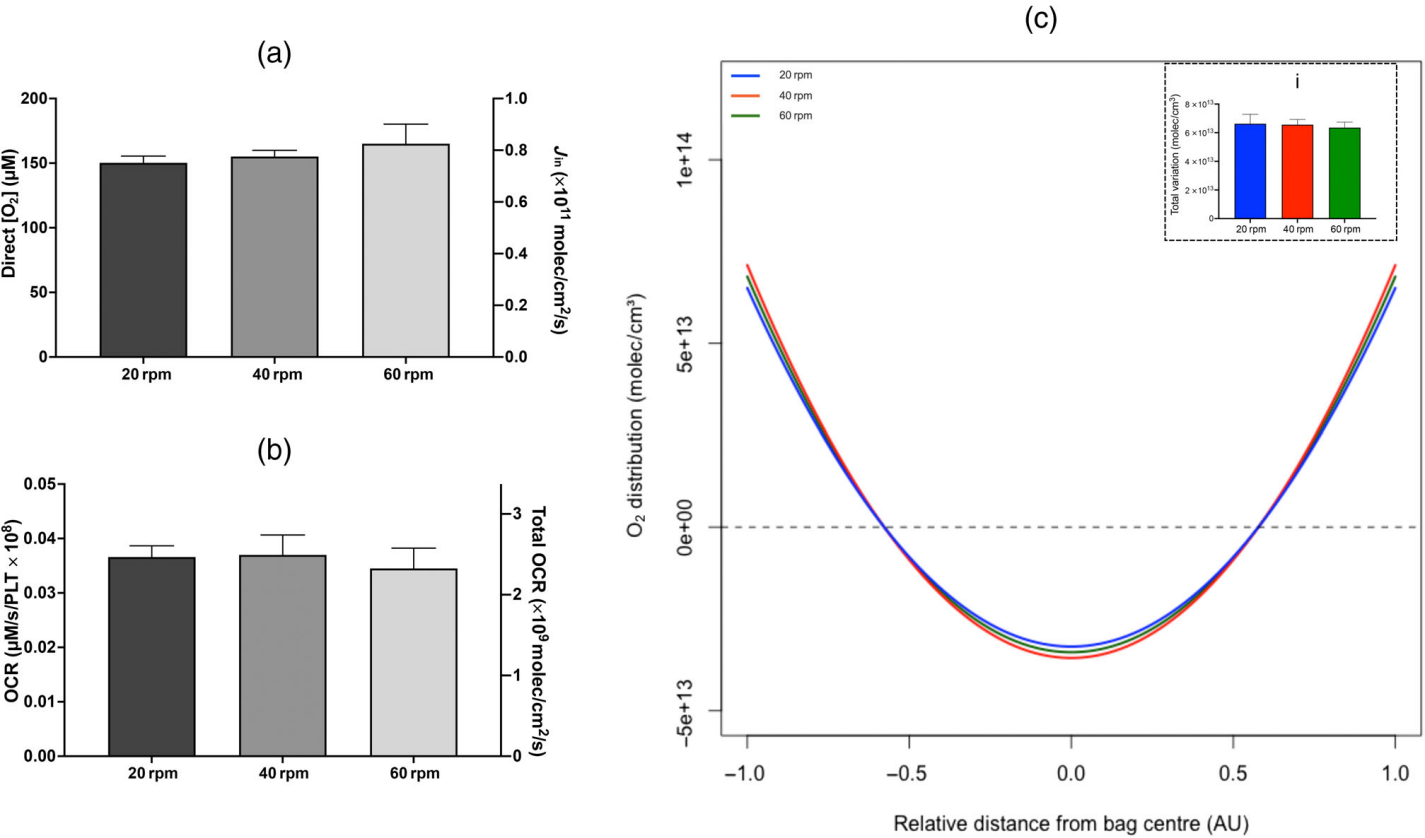
O_2_ availability in neonatal platelet concentrates (PCs) stored at reduced agitation. (a) The measured [O_2_] and calculated *J*_in_. (b) The oxygen consumption rate (OCR) measured by electron paramagnetic resonance (EPR) and the total OCR calculated by Equation (3). (c) The O_2_ distribution, with the *X*‐axis representing the relative location in the bag in arbitrary units (AU), either the bag midpoint (0) or the bag surface (±1). The solid green line shows the O_2_ distribution for neonatal PCs stored at 60 rpm, the solid red line shows the O_2_ distribution for neonatal PCs stored at 40 rpm and the solid blue line shows the O_2_ distribution for neonatal PCs stored at 20 rpm. The dotted black line depicts a homogeneous O_2_ distribution. (i) The total deviation of O_2_ with reduced agitation. Statistical significance was inferred by one‐way analysis of variance (ANOVA) (*N* = 6).

Agitation frequency was shown to have no significant effect on the direct [O_2_] measured in neonatal PCs or on the calculated O_2_ influx (Figure 4a). Reduced agitation frequency also had no significant effect on the measured OCR and the modelled total OCR (Figure 4b). The predicted [O_2_] distribution in neonatal PCs stored at 60 rpm deviates equally from a homogeneous distribution compared to neonatal PCs stored at 20 and 40 rpm (Figure 4c). Comparing the inner edge of the bag to the midpoint, neonatal PCs stored at 60 rpm deviate from homogeny by +4.23 ± 0.256 and −2.12 ± 0.128 molec × 10^13^/cm^3^, respectively; neonatal PCs stored at 40 rpm deviate from homogeny by +4.42 ± 0.438 and −2.21 ± 0.219 molec × 10^13^/cm^3^, respectively; neonatal PCs stored at 20 rpm deviate from homogeny by +4.37 ± 0.244 and −2.19 ± 0.122 molec × 10^13^/cm^3^, respectively (Table 1). Moreover, there was no significant difference in the total deviation of O_2_ throughout the PCs at all agitation rates (Figure 4c, panel i).

**FIGURE 5:**
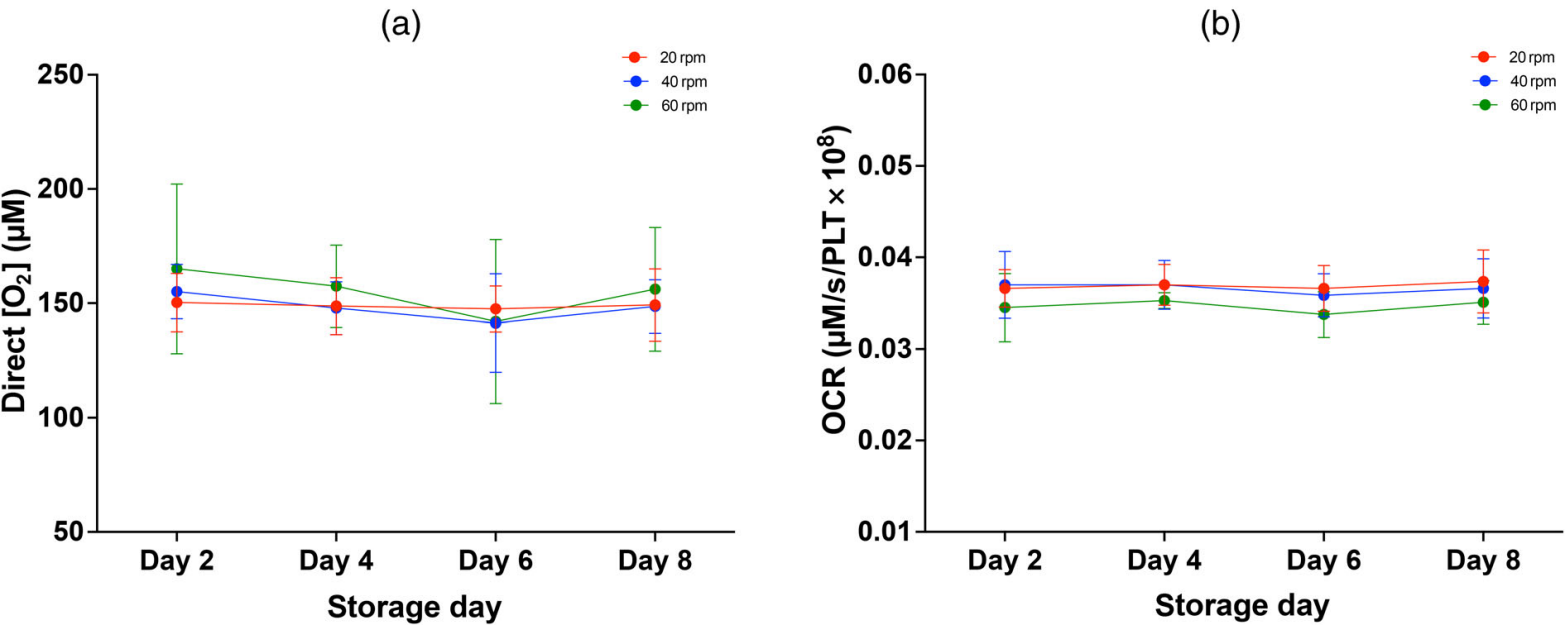
The influence of agitation speed on direct [O_2_] and oxygen consumption rate (OCR) over 8 days of storage. (a) Change in direct [O_2_] and (b) change in OCR over 8 days of storage. Graphs show the mean values with error bars representing SD (*N* = 6). Significance was inferred by a two‐way analysis of variance (ANOVA) followed by a Tukey's post hoc test. Significance is denoted as follows: *p < 0.05.

Over the 8‐day storage duration, no significant difference in the direct [O_2_] measured was observed for neonatal PCs stored at 20, 40 and 60 rpm (Figure 5a). Likewise, the measured OCR remained unchanged over the storage duration for PCs stored at 20, 40 and 60 rpm, and there was no significant difference in OCR between agitation speeds at any point during storage (Figure 5b).

## DISCUSSION

This study provides novel insights into the relationship between [O_2_], OCR and agitation speed in the storage of neonatal PCs. The study demonstrates that neonatal PCs are able to maintain a higher direct [O_2_] compared to adult PCs (Figure 2a) despite a significant reduction in oxygen influx (Figure 2b). Likewise, neonatal PCs are also able to withstand greater insult to their ambient storage [O_2_], in comparison to their adult dose counterparts (Figure 3a). Reducing the stored agitation speed to 20–40 rpm was found to have no significant effect on the O_2_ availability of neonatal PCs over 8 days storage (Figure 5).

These observations are likely attributed to the smaller bag sizes of neonatal PCs and the reduced degree of bag filling relative to the maximum bag capacity (Table 1); ultimately, this would increase the surface area‐to‐volume ratio of the PC and minimize the distance from the bag midpoint to the bag surface in neonatal PCs. These factors can improve oxygenation in several ways. Firstly, the reduced distance from the bag midpoint to the bag surface would limit the total OCR per parallelepiped of the neonatal PC and reduce the summative effect of O_2_ consumed when approaching the midpoint to the bag. Secondly, the decrease in the diffusion distance of O_2_ within the neonatal PC would promote the flux of O_2_ into the PC. Diffusion flux refers to the amount of a substance that diffuses through unit area per unit time [21]. Lastly, a relative increase in the surface area compared to the PC volume would increase the rate of O_2_ diffusion into the PC. In contrast to the diffusion flux, diffusion rate refers to the total amount of substance that diffuses through a system per unit time, and could be considered as follows: *R* = *J* × *s*, where *R* is the rate of diffusion, *J* is the flux and *s* is the surface area. Assuming one‐dimensional steady‐state diffusion, the rate of diffusion is proportional to the diffusive surface area, and the flux of O_2_ is inversely proportional to the diffusive distance (Equation 4); therefore, the flux of O_2_ will increase as the distance required for diffusion decreases, and the rate of diffusion will increase as the surface area increases. These explanations are supported by Figure 2, showing that despite ~1.53‐fold reduction in O_2_ influx, because of the differences in material gas permeability, neonatal PCs can still maintain a significantly higher direct [O_2_] compared to adult PCs as a result of the nearly 3.78‐fold reduction in total OCR.

The above observations are further emphasized when the ambient storage [O_2_] is challenged. The study shows that neonatal PCs stored at 5% [O_2_] are able to maintain an internal [O_2_] sufficient to sustain an OCR equal to that of neonatal PCs stored at 21% [O_2_]. This is expected, as the measured O_2_ influx exceeds that of the total OCR, so the OCR can remain constant [22]. Comparatively, adult PCs stored at 5% [O_2_] showed the measured direct [O_2_] and OCR to diminish, alongside the development of substantial regions of anoxia. Since mitochondrial respiration should proceed at a near‐maximum rate at [O_2_] above 0.08 μM [23], it is unlikely that this measurement was obtained because of mitochondrial dysfunction. Instead, this observation is most likely due to the O_2_ demand at 5% [O_2_] (total OCR) exceeding that of O_2_ influx. This would suggest that the O_2_ influx limits the total OCR in adult PCs stored at 5% [O_2_] but not in neonatal PCs stored at 5% [O_2_]. This conclusion suggests neonatal PCs are tolerant to reduced ambient [O_2_] storage resembling physioxia (~3%–5% [O_2_]) [24].

Lastly, the efficacy of agitation to improve O_2_ availability was challenged. This study showed no change in the direct [O_2_], the O_2_ influx or the total variation of O_2_ throughout the PC at reduced agitation during storage (20–40 rpm) compared to neonatal PCs stored at the current, commonly applied agitation frequency of 60 rpm (Figure 4). This was also seen over 8 days of storage (Figure 5). Although agitation or ‘shaking’ has shown improvements in bacterial cell growth and viability in culture and bioreactors by improving the mass transfer of O_2_ [25], mammalian cells have been shown to require less O_2_ for energy generation because of their reduced growth rate and metabolic activity and, therefore, mammalian cell viability may not be as dependent on agitation frequency for oxygen transfer as aerobic bacterial cultures [26, 27]. A study conducted by Zhang et al. supports this, showing that mammalian cell growth is almost independent of the specific power input (proportional to the shaking frequency of the flask), as the oxygen transfer rates remained higher than the oxygen uptake rate of the cells at all shaking frequencies [25].

Despite this, interrupted agitation during PC storage has previously been shown to alter the biochemical properties such as pH and lactate [9]. Although biochemical measurements were not done in this case, this study suggests that the previous observations are likely due to interrupted agitation being unable to maintain sufficient PLT suspension, leading to contact activation with the bag surface. PLTs exhibit high metabolic plasticity to adapt to the varying energy demands of the cell [28]. Upon activation, the energy requirements of PLTs rise because of activation‐associated contractile events [29, 30]. This increased demand is likely met by shifting towards a glycolytic metabolic phenotype, which subsequently increases lactate production [28]. Therefore, the study proposes that agitation during PC storage is primarily required to maintain PLT suspension and a homogeneous OCR, rather than to promote gaseous exchange across the film.

EPR is a well‐established method to accurately measure [O_2_] and OCR within a sample. Despite this, the method brings some limitations. EPR requires calibration prior to each use, which can vary in duration between samples. This, in conjunction with the closed system used for accurate OCR measurements of samples, can result in a slight delay in the start of recording in the initial sample [O_2_]. To minimize this error, EPR spectra were recorded after a set time had elapsed (1 min). This ensured consistency across all samples and, given the relatively low measured OCR, ensured <5% variation from the measured [O_2_] to the absolute [O_2_]. The study is also limited by a small sample number, which was partially addressed by using a pool‐and‐split study design [19]. This provided some statistical benefits to the study. Pooling multiple donations limited the inter‐donor variability, and splitting provided an opportunity to conduct pairwise analysis.

Moreover, it is important to note the model used to predict O_2_ distribution was based on the assumption of sufficient mixing of PLTs throughout the PC by agitation and therefore did not consider the widely accepted hypothesis of local anoxia induced by regions of denser PLT populations at reduced agitation frequencies or the influence of boundary effects. Likewise, we acknowledge that the absence of agitation was not investigated in this study. However, because of the abundance of O_2_ in both adult and neonatal PCs stored at 21% [O_2_], the O_2_ influx is greater than the total OCR, and because of the predicted near‐homogeneous O_2_ distribution throughout the length of the PC, it is likely that local anoxia cannot exist under the standard blood banking conditions in both adult and neonatal PCs. Moreover, as the total number of PLTs per parallelepiped is constant, local changes in the OCR throughout the PC would have to add up to the total OCR of the parallelepiped; therefore, [O_2_] at the midpoint of the bag would likely be equal in a homogeneous and heterogeneous PLT suspension. Lastly, the model assumes that the PC bag itself has a cuboidal shape, allowing each parallelepiped to be identical and behave similarly; however, in practice, the PC bag has a slight curvature, suggesting that parallelepipeds further from the bag centre may have improved diffusive characteristics, which may have been underrepresented by this model.

Another important consideration when translating the presented O_2_ dynamics to products stored in 100% plasma is the difference in diffusion coefficient of plasma (*D*_plasma_) and water (*D*_water_). The study models O_2_ diffusion using values derived from aqueous media; however, a linear decrease in *D*_plasma_ has been demonstrated as the protein content increases [31]. As such, the reported value for *D*_plasma_ at 25°C is approximately 1.62 × 10^−5^ cm^2^/s, compared to ~2.42 × 10^−5^ cm^2^/s for *D*_water_ [31]. As the PCs examined in this study were stored in a 65:35 PAS/plasma ratio, and given that PAS more closely resembles water compared to plasma, the O_2_ diffusion properties may differ from those of PCs stored in 100% plasma. Nonetheless, while absolute diffusion values may vary between storage media, the overall trends observed in this study—particularly the effects of container surface area, fill volume and material type on oxygen availability—are expected to remain consistent.

This study reinforces previous findings that suggest the commonly used agitation rate of 60 rpm may not be necessary for maintaining [O_2_] when storing PCs in small‐volume containers [6]. Therefore, reduced agitation strategies could be pivotal for improving neonatal PC storage. However, optimizing agitation requires careful consideration, as it needs to minimize agitation‐induced shear stress while maintaining a sufficient cellular suspension. Likewise, our findings suggest the use of larger bags with lower fill volumes, or materials with higher O_2_ transmission rates, can significantly improve O_2_ availability throughout PC storage. Future studies are warranted to systematically assess the optimal bag design, fill volume or material type as well as their impact on PC quality and storage outcomes.

## CONFLICT OF INTEREST STATEMENT

The authors declare no conflicts of interest.

## Supporting information


**Data S1.** Supporting information.

## Data Availability

The findings of this study are included in the article and available in the Supporting Information.
