# Supplementary material for: Enhanced O2 availability in platelet concentrates stored for neonatal transfusion is independent of agitation: Evidence from direct oximetry and Fickian diffusion modelling
Source: Vox Sang. 2025 Aug 28;120(12):1232–41. doi: 10.1111/vox.70101 (PMC12695442; doi:10.1111/vox.70101)
Supplement: Supplementary file 1 — Data S1. Supporting information. [file VOX-120-1232-s001.docx]

**Supplementary material**

Fickian-diffusion model

A one-dimensional approach can be used due to the symmetry of the task, (**Figure S1**). Тhe axis Х is selected from the center plane of the bag. A one-dimensional steady-state diffusion equation of oxygen concentration [O_2_] inside the bag is described as Eq S1.

$\left( S1 \right) \frac{d^{2}\left[ O_{2} \right]}{{dx}^{2}}-\frac{OCR}{D}=0$

Where: $\frac{d^{2}\left[ O_{2} \right]}{{dx}^{2}}$ is the second derivative of oxygen concentration, D is the oxygen diffusion coefficient in the media (assumed equal to water at D=2.42E-5 cm^2^/s)), OCR is oxygen consumption rate. The solution of the Eq S1 presents oxygen concentration distribution inside the element (Figure S1) and can be expressed by Eq. S2.

$\left( S2 \right) \left[ O_{2} \right]=\frac{OCR}{2*D}*x^{2}+C_{1}x+C_{2}$

Where: C_1_ and C_2_ are constants. [O_2_] in the center (x=0) achieves minimum, so the first derivative d[O_2_]/dx=0, and therefore C_1_=0. Integration of the Eq. S2 allows to tie up constant C_2_ and average oxygen concentration <[O_2_]> in the bag (Eq S3).

${\left( S3 \right) C}_{2}=<\left[ O_{2} \right]>-\frac{OCR}{D*6}d^{2}$

Where: d = half of the bag thickness. Finally, the parabola is shown in Eq. S4, where O_2_ represents oxygen concentration at a given position within the PC, ‘OCR’ represents oxygen consumption rate, ‘D’ represents the diffusion coefficient (given as D_water_ at 25°C= 2.42x10^-5^ cm^2^/s) , ‘x’ represents the distance from a certain point in the bag to the center of the bag (excluding film thickness), ‘<[O_2_]>’ represents the direct O_2_ measured, and ‘d’ represents the distance to the center of the bag.

$$\left( S4 \right) O_{2}=\frac{OCR}{2*D}*x^{2}+<\left[ O_{2} \right]>-\frac{OCR}{D*6}d^{2}$$

**
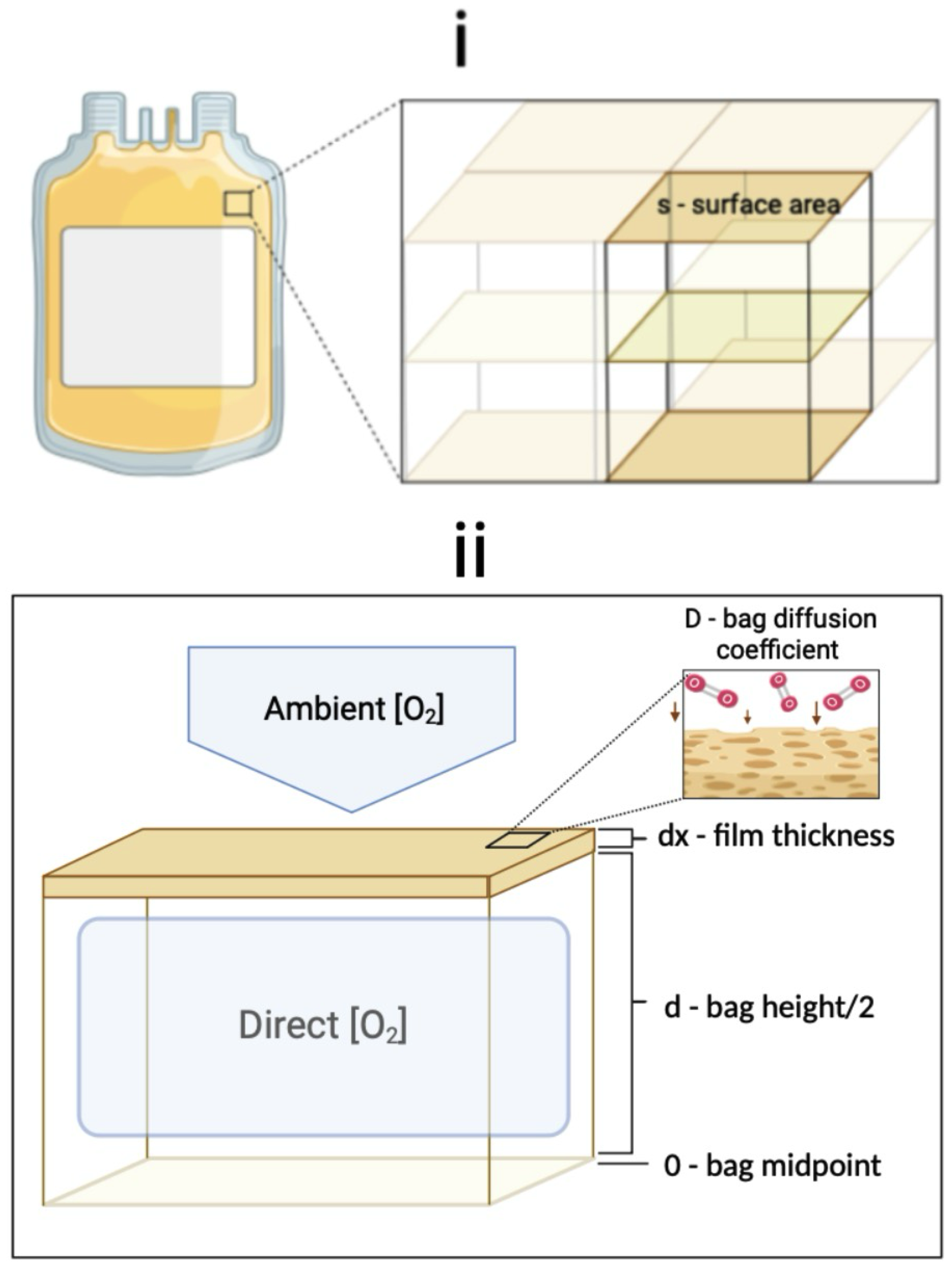
**

**Figure S1. The conceptual basis of the O_2_ modelling of a homogenous suspension**. Illustration (i) displays a cuboidal structure divided into identical parallelepipeds, acting through a 1cm^2^ surface area, s. Each parallelepiped can be split into two segments (ii); one representing the film thickness, dx, and the other, bag height, d. The distance from a certain point to the centre of the bag is measured, x. The ambient [O_2_] refers to the concentration of O_2_ outside of the bag, while the direct O_2_ represents the average [O_2_] inside the bag
